# Direct imaging of the disconnection climb mediated point defects absorption by a grain boundary

**DOI:** 10.1038/s41467-022-29162-2

**Published:** 2022-03-18

**Authors:** Jiake Wei, Bin Feng, Eita Tochigi, Naoya Shibata, Yuichi Ikuhara

**Affiliations:** 1 Institute of Engineering Innovation, The University of Tokyo, Tokyo, 113-8656 Japan; 2 Center for Elements Strategy Initiative for Structural Materials, Kyoto University, Kyoto, 606-8501 Japan; 3 Institute of Industrial Science, The University of Tokyo, Tokyo, 153-8505 Japan; 4 PRESTO, Japan Science and Technology Agency, Saitama, 332–0012 Japan; 5 Nanostructures Research Laboratory, Japan Fine Ceramics Center, Nagoya, 456-8587 Japan

**Keywords:** Surfaces, interfaces and thin films, Transmission electron microscopy

## Abstract

Grain boundaries (GBs) are considered as the effective sinks for point defects, which improve the radiation resistance of materials. However, the fundamental mechanisms of how the GBs absorb and annihilate point defects under irradiation are still not well understood at atomic scale. With the aid of the atomic resolution scanning transmission electron microscope, we experimentally investigate the atomistic mechanism of point defects absorption by a ∑31 GB in α-Al_2_O_3_ under high energy electron beam irradiation. It is shown that a disconnection pair is formed, during which all the Al atomic columns are tracked. We demonstrate that the formation of the disconnection pair is proceeded with disappearing of atomic columns in the GB core, which suggests that the GB absorbs vacancies. Such point defect absorption is attributed to the nucleation and climb motion of disconnections. These experimental results provide an atomistic understanding of how GBs improve the radiation resistance of materials.

## Abstract (web summary)

Grain boundaries can improve the radiation resistance of a material by annihilating point defects formed under irradiation, however the atomistic mechanism is still unclear. Here the authors demonstrate grain boundaries absorb point defects through the climb motion of disconnections.

## Introduction

Materials in nuclear systems must be able to withstand high dose irradiations of high energy electrons, neutrons and ions. Under irradiation, point defects such as self-interstitials and vacancies are significantly produced^1^, in which the continued accumulation of these point defects forms interstitial clusters, stacking fault tetrahedral and voids in the materails^2–6^. These defects would lead to swelling, hardening, amorphization and embrittlement, which finally cause materials failures^6–9^. It has been reported that grain boundaries (GBs) could help withstand the radiation damages^10–14^. Nanocrystaline materials, which contain large fractions of GBs, have better radiation resistances compared with their single crystalline counterpart^12,13^. It is speculated that GBs could serve as effective sinks for the point defects generated under irradiations, i.e., the GBs absorb and annihilate these point defects^11,15–17^. Although such an idea has been well accepted nowadays, the fundamental mechanisms on whether these point defects are indeed annihilated by the GBs and how the GBs evolve after absorbing the point defects are far from clear at atomic scale.

To date, many efforts have been devoted to investigating on the atomistic mechanisms of the point defect-GB interactions^15,18–27^. However, most of these studies were based on theoretical calculations, which are limited to some specific GBs in metals due to the restrictions of the computational resources. On the other hand, recent developments of the transmission electron microscope (TEM) attached with high energy ionic sources enable us to observe the real-time evolutions of materials containing GBs under ionic irradiation^21,23^. However, due to the complexity of GB structures, and the nature of non-straightforward image interpretation in high-resolution TEM, the structural details of GBs are difficult to be fully determined at atomic scale. Therefore, it is still experimentally unclear how the GBs interact with the point defects under irradiation.

Here, we experimentally demonstrate that the point defects could be absorbed and annihilated by the climb motion of the disconnection at a GB in α-Al_2_O_3_, by atomic resolution scanning transmission electron microscope (STEM). A disconnection is a line defect in the GB, which has characters of a step (step height h) and a dislocation (Burgers vector **b**)^28^. We show that a disconnection pair is formed under high energy electron beam irradiation in the vicinity of the GB. After tracking all the Al atomic columns during the formation of a disconnection pair, we demonstrate that it is proceeded with disappearing of Al columns, suggesting the GB absorbs vacancies. Such point defect absorption is attributed to the disconnection climb motion on mesoscopic scale.

## Results

### Formation and characterization of the disconnection pair

**Fig. 1:**
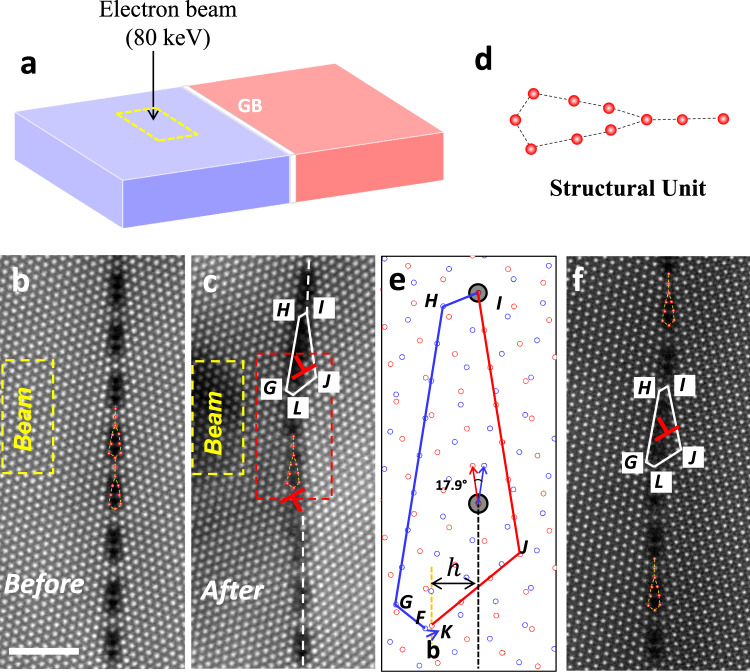
Disconnections formation in the ∑31 GB after electron beam irradiation. **a** Schematical diagram of the experimental setup. An 80 keV high energy electron beam with current of 30 pA was focused to irradiate on one of the grains near the GB in the dotted yellow box area. **b** The HAADF image of the pristine ∑31 GB. The structure unit is overlaid on the image. **c** The HAADF image after scanning the incident electron beam at the dotted yellow box in **a** for 1.5 min. The structure unit at the migration front in the dotted red box is same to the pristine GB, which is overlaid. **d** The structural unit of the pristine GB, which is also overlaid in **b**, **c** and **f**. **e** The dichromatic pattern of the ∑31 GB, in which the blue and red circles denote to the lattice position of Al columns in the left and right crystals respectively, and the larger gray circles are the CSL columns. The upper disconnection in **c** is characterized as $({{{{{\rm{h}}}}}}=3.4\,{{{\AA}}},{{{{{\bf{b}}}}}}=1/300{[16,\bar{19},{{{{\mathrm{3,0}}}}}]}_{{RED}})$ as shown in **e**. **f** The same disconnection found in pristine bicrystal. The scale bar in the images is 2 nm.

α-Al_2_O_3_ is one of the most important oxides, which is considered as a candidate of the structural materials in irradiation environments^10,29,30^. In this study, a coincident site lattice (CSL) ∑31$\left\{7\bar{11}40\right\}/\left[0001\right]$ GB of α-Al_2_O_3_ was chosen and fabricated by thermal bonding of two single crystals^30^, where ∑ represents the degree of geometric coincidence at the GB. According to the CSL theory, the GB with large ∑ value can be classified to a random GB^31^, of which the property of this GB is also comparable to the random distributed GBs in polycrystalline α-Al_2_O_3_^32^, and its atomic structure has been studied in detail^30^. To investigate the point defect interaction with this GB, an 80 keV electron beam with a high probe current (30 pA) in the STEM system was controlled to scan on one of the grains in the vicinity of the GB, as shown in the dotted yellow box in Fig. 1a^33^. Under electron beam irradiation, the point defects could be produced in the bulk region near the GB (See more discussion in Supplementary Note I)^34,35^, which enable us to analyze the potential interactions between GB and point defects generated. After the irradiation, the atomic structural evolution of the GB was systematically imaged by STEM with a low current probe (7 pA) to avoid any unintentional beam damages^33,36^.

Figure 1b shows the high angle annular dark field (HAADF)-STEM image of the pristine ∑31 GB viewing from [0001] direction, of which the GB is in edge-on condition. Bright spots in the image correspond to the Al atomic columns. According to this image, the GB forms periodical structure units along the GB. This GB structure matches well with previous reports^30,37^, where the structural unit was described as several member rings connected. Here, the structural unit is described as a straight line and a polygon for simplicity, which is overlaid on the image and highlighted in Fig. 1d. After scanning the high energy electron beam in the dotted yellow box in Fig. 1b on the left grain for 1.5 min, the GB migrated to the left crystal as shown in Fig. 1c. Similar irradiation induced GB migrations have been observed in other radiation experiments^33,38^, and a detailed comparison will be discussed in the discussion part.

It is important to note that the GB structure at the migration front (shown in the red box of Fig. 1c) is the same as the one in the initial unmoved GB in Fig. 1b, and thus, there is a disconnection pair (two disconnections) formed in the GB between the non-migrated and migrated segments^28^. The dislocation character (i.e., the Burgers vector **b**) of the disconnection pair in the GB can be determined by comparing the Burgers circuit around the disconnection drawn from the experimental image with that obtained from the dichromatic pattern, which is the interpenetrating lattices of both crystals as shown in Fig. 1e. In the experimental image, the circuit around the upper disconnection in Fig. 1c is closed, which is described with IHGL and IJL in the left and right crystals, respectively, and L corresponds to the migration front. When putting the same circuit into the dichromatic pattern in Fig. 1e, the circuit is not closed. The closure vector **FK** is the Burgers vector of this disconnection, which is $1/300{[16,\bar{19},{{{{\mathrm{3,0}}}}}]}_{{RED}}$ in the coordination system of the right (red) crystal (denoted in RED). The size of the Burgers vector ($\left|{{{{{\bf{b}}}}}}\right|$) is 0.5 Å. Meanwhile, the step height (h) is calculated to be 3.4 Å, which is the distance between the unmoved GB (dotted black vertical line across I in Fig. 1e) and the migrated GB (dotted yellow line across L in Fig. 1e). It is worth noting that the same disconnections are also found in another region of the pristine bicrystal, and one example is given in Fig. 1f. This fact indicates that these disconnections should be energetically favorable, which were formed during fabricating the bicrystal at high temperature.

### Column-by-column tracking of the disconnection formation

**Fig. 2:**
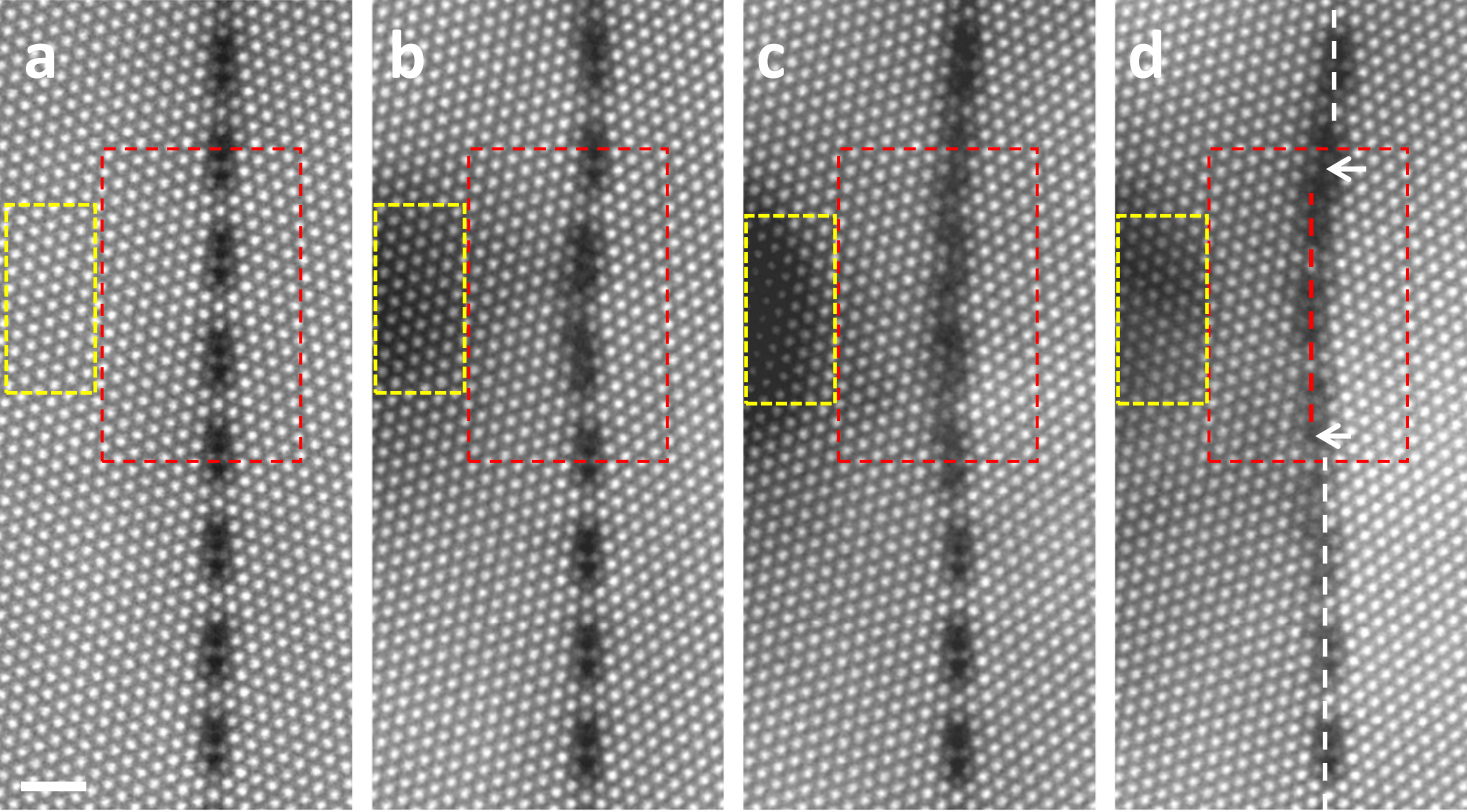
step-by-step imaging of the disconnection formation. The irradiation was controlled step-by-step. For each step in **a**–**c**, the electron beam was focused to scan in the dotted yellow box for 30 s. After three step irradiation induced GB migration in **d**, there are two disconnections formed as highlighted by the white arrows, which are same to Fig. 1c. The migration front in the dotted red boxes will be analyzed column-by-column in Fig. 3. The scale bar in the images is 1 nm.

**Fig. 3:**
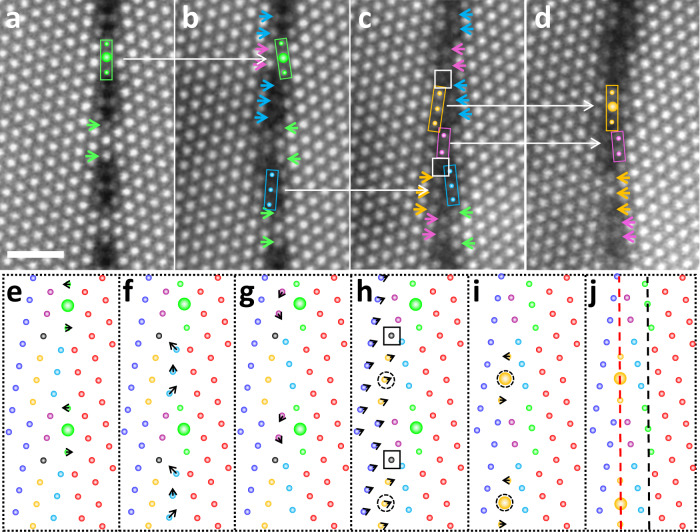
Column-by-column tracking of the disconnection formation. **a**–**d** The enlarged images in the dotted red boxes of Fig. 2. The scale bar is 1 nm. The columns, which are highlighted by left arrows, are displaced to the columns in right arrows with same color in the next image. The green, light-blue, pink, yellow columns or arrows were displaced step-by-step. **e**–**j** The proposed atomic displacements for disconnection formation step-by-step. The spheres are corresponding to the Al columns. The black arrows are the displacements routes on each column. The black and red dotted vertical lines are corresponding to the GB positions before and after electron beam irradiation induced GB migration. In **e**–**g** and **i**, atomic shuffling occurs, and in **h**, the shear process occurs to form the dislocation character of the disconnection pair. The black columns, which are also highlighted by the white boxes in **c** disappeared after the disconnection formation in **d** or **i**, **j**.

**Fig. 4:**
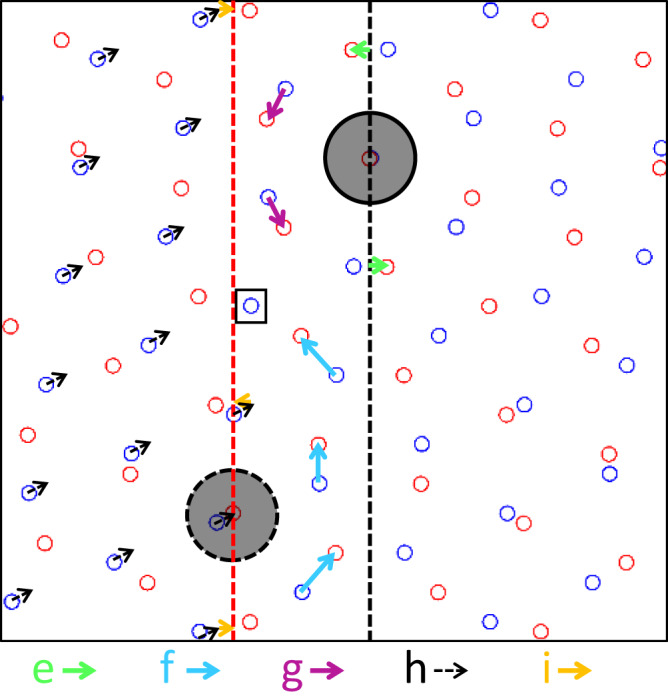
Summarized atomistic disconnection formation into the dichromatic pattern. The blue and red circles are corresponding to the lattice position of the left and right crystal respectively, and the larger solid gray circle is the CSL column before migration. The larger gray dotted circle on the dotted red line is the CSL column at the migration front after shear process. The dotted black and red lines are corresponding to the GB position at pristine and disconnection formation, respectively. The green, light blue, pink and yellow arrows correspond to the atomic shuffling process of the same-colored Al columns in Fig. 3 (**e**, **f**, **g**, and **i**). The dotted black arrows denote to the atomic shear process in Fig. 3h. The blue column in the solid black box disappeared when the disconnection forms.

To further investigate the correlation between the formation of the disconnection pair and the point defects–GB interactions, we tracked all the Al columns during the disconnection formation in Fig. 1c. For this purpose, we divided the formation of the disconnection pair (in Fig. 1c) into several steps. In each step, electron beam was irradiated for 30 s (Fig. 2), so that the GB moves as short as several atomic columns. After each step of irradiation, the atomic structure of the GB was imaged by HAADF-STEM. In this way, we can track the disconnection formation column-by-column. Figure 2 shows one set of such experiments. After three steps of irradiation in Fig. 2d (same to Fig. 1c), the GB structure at the migration front went back to the structure of the initial GB, and the same disconnection pair was formed as highlighted by the white arrows. Enlarged images of the migration fronts inside the red dotted boxes in Fig. 2a–d are given in Fig. 3a–d. Based on these images, the displacements of the Al atomic columns during the formation of the disconnection pair were discussed in Fig. 3e–j, in which the spheres correspond to the Al columns and the black arrows indicate the routes of the displacements on each column. These atomic displacements are also summarized in the dichromatic pattern in Fig. 4 and Supplementary Movie 1. In the following analysis, we will show the atomistic disconnection formation step-by-step in Figs. 3e–j and 4, with the experimental proofs in Fig. 3a–d.

Figure 3a, e show the atomic configurations of the pristine ∑31 GB. After the first-step irradiation, the three green columns, which are vertically aligned in the box of Fig. 3a, inclined and moved to the ledge of the right crystal in Fig. 3b. Detailed analysis shows that the larger middle columns did not move since they are on the lattice positions of both the left and right grains (the CSL columns). The small green columns are displaced along the black arrows shown in Fig. 3e, which are also summarized as the green arrows in the dichromatic pattern in Fig. 4. These atomic displacements are similar to the previous GB migration reports^33,39^. After that, the blue columns in Fig. 3f moved along the black arrows, from the ledges of the left crystal to the ledges of the right crystal. The corresponding experimental proofs are shown as the same-colored spheres or arrows from the left grain in Fig. 3b to the right grain in Fig. 3c. In the similar way, the pink columns in Fig. 3g were displaced from the left crystal to the right crystal, with the experimental observations from Fig. 3b, d. Then in Fig. 3h, as the nucleation of the disconnection pair, the atoms in the left crystal collectively shear by a distance equals to the Burges vector (${{{{{\bf{b}}}}}}=1/300{[16,\bar{19},{{{{\mathrm{3,0}}}}}]}_{{RED}}$) of the disconnection (See more discussion in Supplementary Fig. 1)^28^. Such process is shown as the black arrows in Fig. 3h or the dotted black arrows in Fig. 4. After the shear process, the middle columns in the three yellow spheres, which is highlighted by the dotted circles in Fig. 3h, coincide with the lattice of the right crystal. In this way, these columns become the new CSL column as shown in Figs. 3i and 4. Meanwhile, the atomic columns inside the box in Fig. 3c (Fig. 3h) disappeared during the nucleation of the disconnection. Finally, the two yellow columns in Fig. 3i shuffled to make the GB structure go back to the initial GB structure in Fig. 3j. The corresponding experimental proofs are shown from Fig. 3c to Fig. 3d. It is important to note that the atomic shear displacements in the experimental image of Fig. 3d around the disconnection pair are much more complicated and smaller than the one given in Fig. 3h because of the relaxing of the two disconnections and the interactions between them^28^. Since the Burgers vector of the disconnection pair is extremely small (~0.5 Å), it is not easy to image the shear displacements around the disconnection pair because of the scanning distortion of STEM imaging. Nevertheless, the shear displacements of this disconnection in the pristine bicrystal were experimentally imaged and demonstrated in Supplementary Fig. 1.

It is worth noting that the number of atomic columns is conserved during GB migrations from Fig. 3a, c, and the migration was proceeded by the atom shuffling. The GB migrations here are different from those predicted by theoretical calculations^24–26^, where the GB migrations were considered to be driven by point defects absorption, and the atomic column density of GB is kept changing during migration. In the present case, similar to the previous report^21,33^, it is likely that the GB migrations are driven by the elastic strain produced in the irradiated region. However, there is one Al column in each repeating unit disappeared during the formation of the disconnection pair from Fig. 3c, d, in which the disappeared columns are highlighted by the white and black square in Fig. 3c, h, respectively. Such a fact suggests that the formation of the disconnection pair should absorb vacancies. Actually, as one can see in Fig. 1c, the formation of the disconnection pair and its lateral movements along the GB involve disconnection climb movement, which would absorb and annihilate vacancies^28^. The movement of the disconnections is similar to that of dislocations, but is confined to the GB plane. From another perspective, the absorption of vacancies is associated with the atomic shear process in Fig. 3h, where the dislocation character of the disconnection was nucleated. When the atomic shear process occurs along the Burgers vector to form the disconnections in Fig. 3h, the two crystals would move closely to each other, because the Burgers vector has the component perpendicular to the GB plane. Such movements would increase the atomic density at the GB core. To reduce the atomic density and preserve the pristine ∑31 GB structure, the GB must absorb vacancies, which is consistent with the experimental observations.

## Discussion

It is worth noting that by using the same experimental set up, the migration of a ∑7 GB in α-Al_2_O_3_ was observed recently^33^. Unlike the present result, the migration of the ∑7 GB is proceeded without forming disconnections nor atomic column loss at the GB core. We propose that such difference originates from their different GB characters and structures, which is determined by the energy of potential disconnections. The magnitudes of possible Burgers vectors (the DSC vectors)^28^ of disconnections in the ∑7 GB and ∑31 GB can be geometrically predicted (Supplementary Fig. S2). In the case of ∑7 GB, possible disconnection has large magnitude of Burgers vector. Since the energy of a disconnection is proportional to the square of the magnitude of its Burgers vector, the disconnections with larger Burgers vectors are expected to be energetically unfavorable. Therefore, the ∑7 GB has to avoid forming disconnections during migration, which results in a pure atom shuffling-dominated GB migration without any severe absorption of defects. In the case of ∑31 GB, however, there are several possible disconnections with different Burgers vectors, and the disconnection energies can differ by approximately one order of magnitude (see Supplementary Fig. S2 for discussions), which well explains the GB migration process in this study. When the Burgers vector is large, the GB migrates via atom shuffling without forming disconnections (Fig. 3a–c), which is similar to the case of ∑7 GB. On the other hand, the GB migrates with disconnections when the Burgers vector is small (Fig. 3c, d), and only the disconnection with the smallest Burgers vector is observed because of its smaller disconnection energy. Furthermore, the nucleation of disconnections was accompanied with absorption of extra vacancies. It is most likely that the defects absorbed by the GB were generated under electron beam irradiation in the irradiated grain. Since it is still difficult to image such point defects dynamical process by electron microcopy, interstitials/vacancies absorption and emission might occur during this process depending on the defect formation and migration velocity^15^. Nevertheless, our results directly demonstrated that the formation and climb motion of disconnection at the GB could absorb extra defects, which provides atomistic insight into one of the representative dynamic point defect-GB interaction. These results further indicate that GBs with small magnitudes of Burgers vector tend to generate disconnections during migration, and the nucleation and motion of disconnections might absorb extra point defect, and thus, contribute more to the improvement of radiation resistance.

In summary, the present experiment illustrated that a disconnection pair is formed in the ∑31 GB of α-Al_2_O_3_ under high energy electron beam irradiation in the vicinity of the GB, and directly imaged the formation of the disconnection pair column-by-column by STEM. We demonstrated that the nucleation of this disconnection pair involves climb motion, which absorbs and annihilates vacancies in the grain. These observations should improve our fundamental understandings of GB-point defect interactions and the mechanism of the improvement of the radiation resistance by GB engineering^15^. Meanwhile, the experimental observations of disconnection nucleation and motion would also help understand the atomistic mechanism of GB dynamics^28^ such as GB migration and GB sliding. Moreover, our experimental method, which takes the advantages of both the irradiation environment and atomic resolution structure characterization abilities of STEM, could be applicable to further clarify radiation influences on the atomic structure and properties, such as the radiation creep and radiation-induced GB segregation^40^ in a wide range of materials.

## Methods

The α-Al_2_O_3_ bicrystal containing ∑31 GB was fabricated by thermal diffusion bonding of two α-Al_2_O_3_ single crystals at 1500 °C for 10 h in air. TEM specimens were prepared by mechanical polishing and Ar-ion beam milling. The atomic structures of the GBs were observed using STEM (ARM200CF, JEOL Co. Ltd) operated at 80 kV, with an electron probe current of ~7 pA. The convergence semi-angle of the electron probe was 24 mrad and the scattered electrons were collected using an annular detector spanning the range 90–200 mrad for HAADF imaging. All the images here were acquired on the [0001] zone axis of α-Al_2_O_3_. The thickness of the sample is around 20 nm, which is estimated by the electron energy loss spectroscopy. The sample is thin enough so that the entire Al columns migrated homogeneously along the thickness direction, and a disconnection with length equal to the sample thickness is formed. To drive the point defects-GB interaction, 80 kV electron beam with a probe current of ~30 pA was controlled to irradiate the sample. All the experiments were conducted at room temperature, and the temperature change due to the electron beam irradiation under present experimental conditions is small enough to be negligible^33^.

## Supplementary information

Supplementary Information

Description of Additional Supplementary Files

Supplementary Movie 1

## Data Availability

The data that support the findings of this study are available within the article and the Supplementary Information. Any other relevant data are also available upon reasonable request from the corresponding authors.

## Code availability

Additional data including the codes are available from the corresponding author upon reasonable request.

## References

[1] Watkins GD (1974). EPR observation of close Frenkel pairs in irradiated ZnSe. Phys. Rev. Lett..

[2] Wirth BD (2007). How does radiation damage materials?. Science.

[3] Arakawa K (2007). Observation of the one-dimensional diffusion of nanometer-sized dislocation loops. Science.

[4] Matsukawa Y, Zinkle SJ (2007). One-dimensional fast migration of vacancy clusters in metals. Science.

[5] Uberuaga BP, Hoagland RG, Voter AF, Valone SM (2007). Direct transformation of vacancy voids to stacking fault tetrahedra. Phys. Rev. Lett..

[6] Diaz de la Rubia T (2000). Multiscale modelling of plastic flow localization in irradiated materials. Nature.

[7] Victoria M (2000). The microstructure and associated tensile properties of irradiated fcc and bcc metals. J. Nucl. Mater..

[8] Odette GR, Alinger MJ, Wirth BD (2008). Recent developments in irradiation-resistant steels. Annu. Rev. Mater. Res..

[9] Sickafus KE (2007). Radiation-induced amorphization resistance and radiation tolerance in structurally related oxides. Nat. Mater..

[10] Clinard FW, Hurley GF, Hobbs LW (1982). Neutron irradiation damage in MgO, Al2O3 and MgAl2O4 ceramics. J. Nucl. Mater..

[11] Nita N, Schaeublin R, Victoria M (2004). Impact of irradiation on the microstructure of nanocrystalline materials. J. Nucl. Mater..

[12] Rose M, Balogh AG, Hahn H (1997). Instability of irradiation induced defects in nanostructured materials. Nucl. Instrum. Methods Phys. Res. Sect. B.

[13] Shen TD (2007). Enhanced radiation tolerance in nanocrystalline MgGa2O4. Appl. Phys. Lett..

[14] Fu C-C, Torre JD, Willaime F, Bocquet J-L, Barbu A (2005). Multiscale modelling of defect kinetics in irradiated iron. Nat. Mater..

[15] Bai X-M, Voter AF, Hoagland RG, Nastasi M, Uberuaga BP (2010). Efficient annealing of radiation damage near grain boundaries via interstitial emission. Science.

[16] Ackland G (2010). Controlling radiation damage. Science.

[17] Demkowicz MJ, Hoagland RG, Hirth JP (2008). Interface structure and radiation damage resistance in Cu-Nb multilayer nanocomposites. Phys. Rev. Lett..

[18] Frolov T, Olmsted DL, Asta M, Mishin Y (2013). Structural phase transformations in metallic grain boundaries. Nat. Commun..

[19] Zhang X (2018). Radiation damage in nanostructured materials. Prog. Mater. Sci..

[20] Vattré A, Jourdan T, Ding H, Marinica MC, Demkowicz MJ (2016). Non-random walk diffusion enhances the sink strength of semicoherent interfaces. Nat. Commun..

[21] Li J (2015). In situ study of defect migration kinetics and self-healing of twin boundaries in heavy ion irradiated nanotwinned metals. Nano Lett..

[22] Chen Y (2015). Damage-tolerant nanotwinned metals with nanovoids under radiation environments. Nat. Commun..

[23] Yu KY (2013). Removal of stacking-fault tetrahedra by twin boundaries in nanotwinned metals. Nat. Commun..

[24] Han J, Vitek V, Srolovitz DJ (2015). The interplay between grain boundary structure and defect sink/annealing behavior. IOP Conf. Ser. Mater. Sci. Eng..

[25] Yu WS, Demkowicz MJ (2015). Non-coherent Cu grain boundaries driven by continuous vacancy loading. J. Mater. Sci..

[26] Frolov T (2018). Grain boundary phases in bcc metals. Nanoscale.

[27] Zhu Q (2020). In situ atomistic observation of grain boundary migration subjected to defect interaction. Acta Mater..

[28] Han J, Thomas SL, Srolovitz DJ (2018). Grain-boundary kinetics: a unified approach. Prog. Mater. Sci..

[29] García Ferré F (2016). Radiation endurance in Al2O3 nanoceramics. Sci. Rep..

[30] Buban JP (2006). Grain boundary strengthening in alumina by rare earth impurities. Science.

[31] Randle, V. *The role of the coincidence site lattice in grain boundary engineering* (Institute of Materials, London, 1996).

[32] Matsudaira T, Kitaoka S, Shibata N, Nakagawa T, Ikuhara Y (2011). Mass transfer through a single grain boundary in alumina bicrystals under oxygen potential gradients. J. Mater. Sci..

[33] Wei J (2021). Direct imaging of atomistic grain boundary migration. Nat. Mater..

[34] Egerton RF, Li P, Malac M (2004). Radiation damage in the TEM and SEM. Micron.

[35] Nan J (2016). Electron beam damage in oxides: a review. Rep. Prog. Phys..

[36] Wei J (2020). Direct measurement of electronic band structures at oxide grain boundaries. Nano Lett..

[37] Guhl H (2015). Structural and electronic properties of Σ7 grain boundaries in α-Al2O3. Acta Mater..

[38] Li N (2013). Incoherent twin boundary migration induced by ion irradiation in Cu. J. Appl. Phys..

[39] Gleiter H (1969). The mechanism of grain boundary migration. Acta Metall..

[40] Wang X (2020). Radiation-induced segregation in a ceramic. Nat. Mater..

