# Supplementary material for: Direct imaging of the disconnection climb mediated point defects absorption by a grain boundary
Source: Nat Commun. 2022 Mar 18;13:1455. doi: 10.1038/s41467-022-29162-2 (PMC8933398; doi:10.1038/s41467-022-29162-2)
Supplement: Supplementary file 1 — Supplementary Information [file 41467_2022_29162_MOESM1_ESM.pdf]

Supplementary Materials for

Direct imaging of the disconnection climb mediated point defects absorption by a grain boundary

Jiake Wei[1,2], Feng Bin[1], Eita Tochigi[1], Naoya Shibata[1, 3] and Yuichi Ikuhara[1, 2, 3]*

[1] Institute of Engineering Innovation, The University of Tokyo, Tokyo 113-8656, Japan.

[2] Center for Elements Strategy Initiative for Structural Materials, Kyoto University, Kyoto 606-8501, Japan.

[3] Nanostructures Research Laboratory, Japan Fine Ceramics Center, Nagoya 456-8587, Japan.

*Correspondence to: Y. I. (ikuhara@sigma.t.u-tokyo.ac.jp)

Inventory of Supporting Information:

1. Supplementary Note I: Discussion on the electron beam effect in Al_2O_3
2. Supplementary Figures and Figure Legends
3. Supplementary References

1. Supplementary Note I: Discussion on the electron beam effect on α-Al_2O_3

The electron beam irradiation effect is a complex process, which damages the samples from several mechanisms, including knock-on, surface sputtering, beam induced heating, radiolysis and beam induced electric field[1,2]. For the electron beam energy of 80 keV and probe current of 30 pA on α-Al_2O_3, the knock-on damage and beam induced heating could be neglected[3]. The main damages come from the radiolysis and beam induced electric field, which are discussed below:

Radiolysis

Radiolysis has been suggested to be pronounced in alumina[4]. For alumina, the atomistic process of this damage was suggested to occur via the Knotek-Feibelman mechanism[1,4,5]: the O ions are suggested be positively charged due to the Auger decay and these positive charged O atoms are repelled by the surrounding metal ions and ejected out from the irradiation areas, which may produce O vacancies and/or interstitials.

Beam induced electric field

When the sample is under electron beam, the irradiated area might be positively charged because of the emission of the secondary and/or Auger electrons[1,2]. When the positively charges in the sample cannot be quickly neutralized by electron conducting (most likely in electron insulating materials like Al_2O_3), ionic displacements would occur under the Coulomb forces, which induce the electron beam damage. It has been suggested that the cations with positive charges, such as Al^{3+} in our case, might be repelled out from the irradiation area which are positively charged[2]. These cations ions would either eject to the vacuum or into the nearby bulk to become cation interstitials and leave the sample in the irradiation area enrich of vacancies.

Therefore, in our experiment, radiolysis and beam induced electric field should account for the beam damage, in which point defects and their clusters or aggregations (e.g. void) at and near the irradiation areas might be generated.

2. Supplementary Figures and Legends

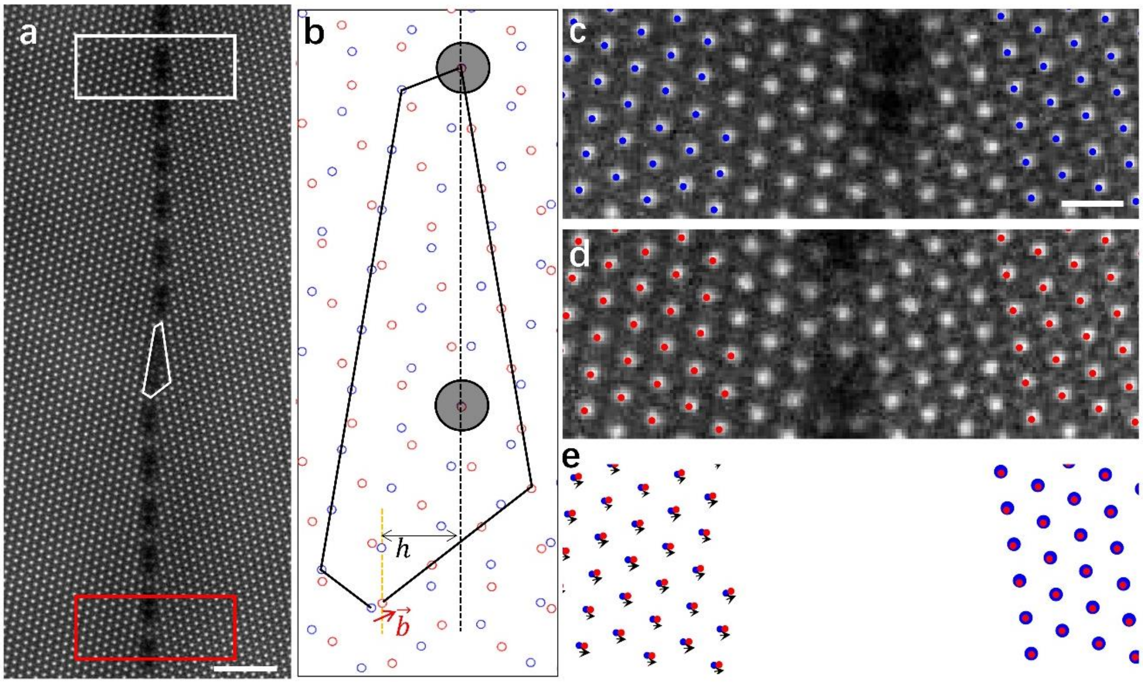

Supplementary Figure 1. Atomic shear displacement of the disconnection. a. The high angle annular dark field image of the disconnection in pristine bicrystal. The scale bar is 2 nm. The image is taken by aligning and averaging of 30 frames, in which each frame was quickly acquired (2.5μs/pixel×1024×512pixels) by the ARM 300CF to improve the spatial resolution and reduce the scanning distortions[6]. **b.** The dichromatic pattern of the GB, which shows the Burges vector of the disconnection is same to the one in Fig. 1c in the main text. **c, d.** The enlarged images in the white and red boxes in **a**, which are before and after the disconnection, respectively. The scale bar is 5Å. The two images are selected to have identical positions for the right crystal. The Al atomic positions are denoted by the blue and red dots, which are located by 2-deminsional Gaussian fitting of the intensity of the image. **e.** The overlaid of atomic column positions in **c** and **d**. The Al columns in the right crystal are set to be coincident. It is clear shown that the Al columns in the left crystal are displaced from the bule dots to the red dots, which indicates when the GB moves, there will be collective shear displacements. These displacements have same direction to the Burgers vector of the disconnection, which are corresponding to the atomic shear displacements in Fig. 3h of the main text.

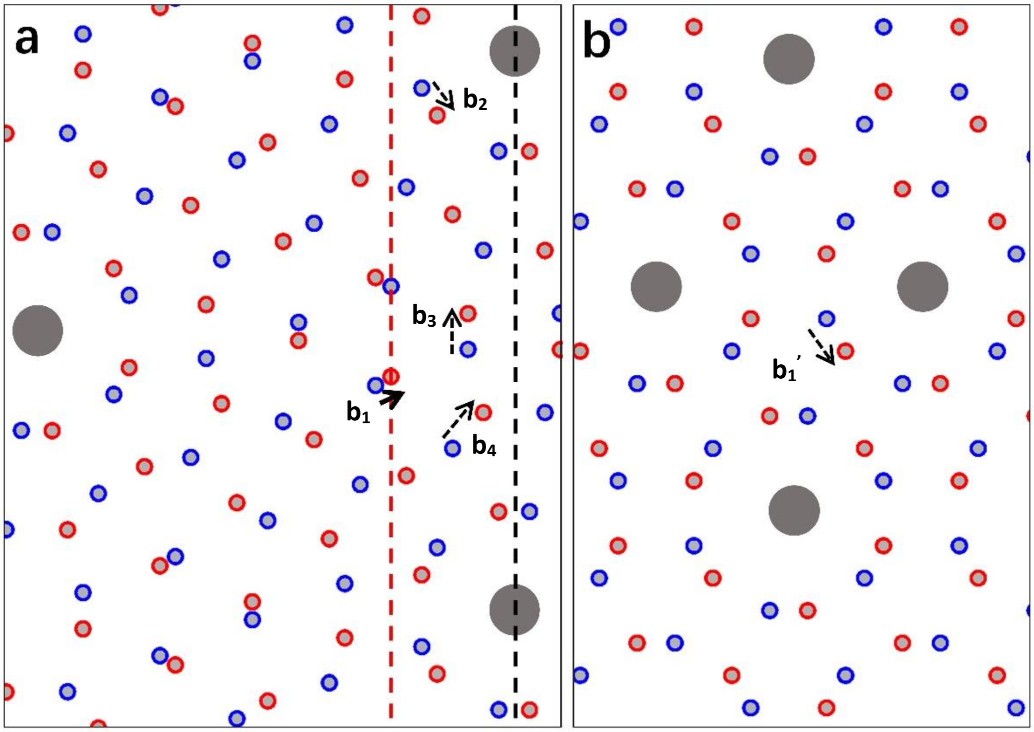

Supplementary Figure 2. The comparison of potential disconnections between ∑31 GB and the ∑7 GB[3]. a. The dichromatic pattern of the ∑31 GB. The blue and red dots correspond to the Al columns in the left and right grain, respectively. The larger grey circles are the CSL columns. The dotted vertical black and red line are the GB positions in the pristine GB and where the disconnection forms, which is also shown in Fig. 4 of the main text. The Burges vector of the disconnection observed in experiment is shown as the solid black arrow on the red line, of which $\mathbf{b_1}$ is about 0.5 Å. When the GB moves from the dotted black line to the red line, there are other disconnection candidates, among which three Burges vector candidates are highlighted by the dotted black arrows. The sizes of the three vectors are $\mathbf{b_2}$=0.88 Å, $\mathbf{b_3}$=0.99 Å and $\mathbf{b_4}$=1.33 Å, respectively. According to the dislocation theory[7], the dislocation energy $E \propto \mathbf{b}^2$, where b is the Burgers vector, and the relative disconnection energies can be estimated as E_{b2}= 3.10E_{b1}, E_{b3}= 3.92E_{b1}, E_{b4}= 7.08E_{b1}. Therefore, only $\mathbf{b_1}$ is observed because it has much smaller disconnection energies compared with all the other disconnections. **b.** The dichromatic pattern of the ∑7 GB. The shortest possible Burgers vector candidate $\mathbf{b_1}'$ (the DSC vector) is highlighted by the dotted black arrow. The size of the potential Burgers vector is 1.04 Å, but the disconnection energy is still much higher than that of $\mathbf{b_1}$ in the ∑31 GB ($E_{b1'}$ = 4.33E_{b1}). Therefore, the

disconnections in the ∑7 GB should be also energetically unfavorable, which have not been found during the GB migration nor in the pristine bicrystal.

3. Supplementary References.

1 Egerton, R. F., Li, P. & Malac, M. Radiation damage in the TEM and SEM. *Micron* **35**, 399-409 (2004).

2 Nan, J. Electron beam damage in oxides: a review. *Rep. Prog. Phys.* **79**, 016501 (2016).

3 Wei, J. *et al.* Direct imaging of atomistic grain boundary migration. *Nat. Mater*. **20**, 951-955 (2021) (2021).

4 Bonevich, J. E. & Marks, L. D. Electron radiation damage of α-alumina. *Ultramicroscopy* **35**, 161-166 (1991).

5 Bouchet, D. & Colliex, C. Experimental study of ELNES at grain boundaries in alumina: intergranular radiation damage effects on Al-L23 and O-K edges. *Ultramicroscopy* **96**, 139-152.

6 Ishikawa, R., Lupini, A. R., Findlay, S. D. & Pennycook, S. J. Quantitative Annular Dark Field Electron Microscopy Using Single Electron Signals. *Microsc. Microanal.* **20**, 99-110 (2013).

7 Anderson,P., Hirth, J., Lothe, J. Theory of Dislocations (3rd Edition). Cambridge University Press (2017)
