# Supplementary material for: Direct imaging of the disconnection climb mediated point defects absorption by a grain boundary
Source: Nat Commun. 2022 Mar 18;13:1455. doi: 10.1038/s41467-022-29162-2 (PMC8933398; doi:10.1038/s41467-022-29162-2)
Supplement: Supplementary file 2 — Description of Additional Supplementary Files [file 41467_2022_29162_MOESM2_ESM.pdf]

Description of Additional Supplementary Files

Supplementary Movie 1. Schematic movie for the atomistic disconnection formation. All these atoms are overlaid on the dichromatic patterns of the ∑31 GB. In the pattern, the blue and red circles denote to the unrelaxed Al columns position in left and right crystals, respectively. The misorientation angle is 17.9° between [1-100] directions of each crystal, corresponding to the orientation relationship of ∑31 GB. The bigger black circles denote to the CSL columns. The spheres on the dichromatic pattern denote to the Al columns, and the red and blue spheres are assigned as columns belong to right and left crystal, respectively.
